# Supplementary material for: Marcus Cross-Relationship Probed by Time-Resolved CIDNP
Source: Int J Mol Sci. 2023 Sep 8;24(18):13860. doi: 10.3390/ijms241813860 (PMC10530771; doi:10.3390/ijms241813860)
Supplement: Supplementary file 1 [file ijms-24-13860-s001.zip › ijms-2577092-supplementary.pdf]

Supplementary Information.

Concentrations and pH used in experiments Q + DP.

quencher	t, °C	pH	C(DP), mM	C(Q), mM
$GMP(-H)^-$	8	11.34	15	9
		11.31	15	18
	25	11.32	15	7.5
		11.40	15	15
	45	11.38	15	6.5
		11.37	15	13
	65	11.39	15	4 (d8-DP)
		11.35	15	8 (d8-DP)
$NacTyrO^-$	8	11.72	15	4
		11.74	15	8
	25	11.71	15	3
		11.74	15	6
	45	11.76	15	2.5
		11.73	15	5
	65	11.73	15	2.5
		11.76	15	5
$TyrO^-$	8	11.76	15	4
		11.76	15	6.5
	15	11.73	15	4
		11.78	15	6.5
	25	11.70	15	3
		11.85	15	6
	35	11.69	15	3
		11.69	15	6
	45	11.73	15	3
		11.75	15	6
	55	11.77	15	2
		11.76	15	3.25
		11.74	15	4.5
	65	11.75	15	1.5
		11.71	15	2.5
		11.72	15	3.5

$GMP(-H)^-$ + $TyrO^-$ + DP

t, °C	pH	C(DP), mM	C($GMP(-H)^-$), mM	C($TyrO^-$), mM
25	11.78	15	4	2.5
	11.73	15	4	1.3
